# Clinical evaluation of vertebral body replacement of carbon fiber–reinforced polyetheretherketone in patients with tumor manifestation of the thoracic and lumbar spine

**DOI:** 10.1007/s00701-023-05502-z

**Published:** 2023-02-23

**Authors:** Maximilian Schwendner, Sebastian Ille, Jan S. Kirschke, Denise Bernhardt, Stephanie E. Combs, Bernhard Meyer, Sandro M. Krieg

**Affiliations:** 1 Department of Neurosurgery, School of Medicine, Klinikum Rechts Der Isar, Technische Universität München, Ismaninger Str. 22, 81675 Munich, Germany; 2 TUM Neuroimaging Center, School of Medicine, Klinikum Rechts Der Isar, Technische Universität München, Ismaninger Str. 22, 81675 Munich, Germany; 3 Department of Diagnostic and Interventional Neuroradiology, School of Medicine, Klinikum Rechts Der Isar, Technische Universität München, Ismaninger Str. 22, 81675 Munich, Germany; 4 Department of Radiation Oncology, School of Medicine, Klinikum Rechts Der Isar, Technische Universität München, Ismaninger Str. 22, 81675 Munich, Germany; 5 Deutsches Konsortium Für Translationale Krebsforschung (DKTK), Partner Site Munich, Munich, Germany; 6 Department of Radiation Sciences (DRS), Institute of Radiation Medicine (IRM), Ingolstädter Landstraße 1, Neuherberg, Germany

**Keywords:** Polyetheretherketone, Dorsoventral instrumentation, Vertebral body replacement, Spinal metastases, En-bloc resection

## Abstract

**Purpose:**

Radiolucent anterior and posterior implants by carbon fiber–reinforced polyetheretherketone (CFR PEEK) aim to improve treatment of primary and secondary tumors of the spine during the last years. The aim of this study was to evaluate clinical and radiological outcomes after dorsoventral instrumentation using a CFR PEEK implant in a cohort of patients representing clinical reality.

**Methods:**

A total of 25 patients with tumor manifestation of the thoracic and lumbar spine underwent vertebral body replacement (VBR) using an expandable CFR PEEK implant between January 2021 and January 2022. Patient outcome, complications, and radiographic follow-up were analyzed.

**Results:**

A consecutive series aged 65.8 ± 14.7 (27.6–91.2) years were treated at 37 vertebrae of tumor manifestation, including two cases (8.0%) of primary tumor as well as 23 cases (92.0%) of spinal metastases. Overall, 26 cages covering a median of 1 level (1–4) were implanted. Duration of surgery was 134 ± 104 (65–576) min, with a blood loss of 792 ± 785 (100–4000) ml. No intraoperative cage revision was required. Surgical complications were reported in three (12.0%) cases including hemothorax in two cases (one intraoperative, one postoperative) and atrophic wound healing disorder in one case. In two cases (8.0%), revision surgery was performed (fracture of the adjacent tumorous vertebrae, progressive construct failure regarding cage subsidence). No implant failure was observed.

**Conclusion:**

VBR using CFR PEEK cages represents a legitimate surgical strategy which opens a variety of improvements—especially in patients in need of postoperative radiotherapy of the spine and MRI-based follow-up examinations.

**Supplementary Information:**

The online version contains supplementary material available at 10.1007/s00701-023-05502-z.

## Introduction

Over the past decades, great advances in tumor treatment have been accomplished. This leads to an exponential rise in the incidence of metastatic cancer including manifestations in the spine. Bone is the third most common site for metastasis after the lung and liver with the spine being the most common region for skeletal metastases [3, 16]. An interdisciplinary management for optimal patient treatment including chemotherapy and immunotherapy, radiotherapy, and surgical treatment is crucial [5, 18]. Surgery is indicated in cases of mechanic instability, spinal cord compression, and severe pain as well as for oncological indications such as biopsy for histopathological analysis and tumor resection. In cases of extensive tumor manifestation or planned total vertebrectomy, anterior tumor removal and dorsoventral instrumentation including vertebral body replacement (VBR) are mandatory [2, 4]. For traumatic or osteoporotic fractures, titanium implants are routinely being used at multiple centers worldwide. However, titanium leads to artifacts on postoperative imaging impairing postoperative adjuvant radiotherapy planning and radiological follow-up to rule out tumor progression especially at the surgical site.

Until today, there have been considerable attempts to optimize surgical treatment enabling improved imaging for follow-up. In the last years, radiolucent anterior and posterior implants by carbon fiber–reinforced polyetheretherketone (CFR PEEK) entered the market allowing for superior radiotherapy planning and follow-up imaging. Regarding dorsal instrumentation, previous studies showed that CFR PEEK pedicle screws improved postoperative imaging quality and radiotherapy dose calculation accuracy while maintaining a risk profile and implant failure rates comparable to conventional metallic instrumentation [14, 19, 21]. Yet, analysis especially of anterior CFR PEEK implants in tumor surgery remains sparse.

The aim of this study was to examine the clinical and radiological outcome of patients with tumor manifestation of the thoracic and lumbar spine treated with vertebral body replacement using CFR PEEK implants.

## Methods

### Hypothesis

Our hypothesis is that the use of CFR PEEK cages for (VBR) is a feasible and safe surgical treatment, especially for patients requiring adjuvant cancer treatment.

### Study protocol

Patients with tumor manifestation of the thoracolumbar spine treated with dorsoventral instrumentation including a CFR PEEK VBR were considered eligible for this study and retrospectively included. Subsequently, patients undergoing dorsal instrumentation only or VBR using a titanium-based cage were not included in this study. Perioperative complications requiring medical attention regarding further treatment and diagnostics were analyzed during the whole inpatient stay. Cage positioning was analyzed postoperatively as well as at follow-up examinations.

### Surgical procedure

Indications for the operative procedure were discussed at our neurosurgical department meeting consisting of seven experienced neurosurgeons with spinal focus. Surgical experience regarding indication and operative procedures was persistent over recruitment time. Dorsal instrumentation was routinely performed with navigated CFR PEEK pedicle screw (Vader®, Icotec AG, Altstaetten, Switzerland) instrumentation two segments above and two segments below the diseased vertebrae. No hooks or wires were implanted. VBR surgery was performed mostly via an open lateral retroperitoneal or transthoracic approach depending on the level of surgery. In two patients with tumor manifestation of the upper thoracic spine, cage implantation was performed via dorsolateral approach and costotransversectomy. For lateral retroperitoneal or transthoracic approach, the patient was rotated in a 90° lateral position using a vacuum mat molded to the body. No access surgeon was required. In cases of navigated cage placement, a reference array was attached to the pelvis, three-dimensional (3D) imaging was performed intraoperatively, and spinal navigation was applied throughout the surgery for skin incision planning, surgical preparation, and implant placement. An expendable CFR PEEK implant (Kong®, Icotec AG, Altstaetten, Switzerland) was placed under guidance of neuronavigation or X-ray in lateral and anterior–posterior orientation after the vertebrae and adjacent vertebral discs were resected. The size of the body and endplates of the implant were determined on preoperative imaging and verified using intraoperative test implants. Intraoperative cage expansion was performed until the cage showed proper positioning and retaining force. This procedure does not differ from the routine procedure in the case of the application of titanium implants.

### Radiographic analysis

Imaging preoperatively, intraoperatively, and at follow-up examinations were reviewed for radiographic analysis. Regarding sagittal correction, the anterior and posterior height of the interspace between the lower endplate of the vertebrae above and the upper endplate of the vertebrae below the diseased level were measured. The resulting angle between the upper endplate of the vertebrae above and upper endplate of the vertebrae below of the diseased level in a sagittal view was acquired in addition. Cage placement was evaluated in sagittal and anterior–posterior direction. Measurements were performed on preoperative and postoperative CT scans as well as on CT scans at follow-up.

### Data analysis

Statistical analyses were performed using Prism (version 8.4.1; GraphPad Software, La Jolla, CA, USA). Descriptive statistics including mean, median, minimum, maximum, and standard deviation were calculated for patient- and fracture-related characteristics including radiographic measurements.

## Results

### Patient characteristics

A consecutive series of 25 patients (8 women, 17 men) aged 65.8 ± 14.7 (27.6–91.2) years with 37 vertebrae of tumor manifestation requiring dorsoventral instrumentation of the thoracic and lumbar spine were analyzed. Median ASA-PS (American Society of Anaesthesiologists physical status) class was 3 (1–4). Symptoms at diagnosis were pain (88.0%), neurological deficits (32.0%) such as spinal ataxia (20.0%) or sensory-motor deficits (12.0%), and asymptomatic progressive spinal metastasis at follow-up examinations (4.0%) (Table 1). Two cases (8.0%) of primary spinal tumor (chondrosarcoma and aneurysmal bone cyst) as well as 23 cases (92.0%) of spinal metastases were treated (Table 1).

**Table 1:** Patient data and risk factors

***n* (%)**	
**Number of patients**	25
**Female gender**	8 (32.0)
**Age at surgery (y; mean ± SD; range)**	65.8 ± 14.7 (27.6–91.2)
**ASA-PS class**	
**Median** • Class 1 • Class 2 • Class 3 • Class 4	3 0 (0.0) 7 (28.0) 16 (64.0) 2 (8.0)
**Symptoms at diagnosis**	
• Pain • Neurological deficits - Spinal ataxia - Sensory-motor deficits • Asymptomatic (follow-up)	22 (88.0) 8 (32.0) 5 (20.0) 3 (12.0) 1 (4.0)
**Tumor**	
• Prostate cancer • Lung cancer • Breast cancer • Myeloma • Adeno carcinoma • Renal cell carcinoma • Urothelial carcinoma • Chondrosarcoma • Primitive neuroectodermal tumor • Lymphoma • Malignant melanoma • Aneurysmal bone cyst	4 (16.0) 3 (12.0) 3 (12.0) 3 (12.0) 2 (8.0) 2 (8.0) 2 (8.0) 2 (8.0) 1 (4.0) 1 (4.0) 1 (4.0) 1 (4.0)

Table 1 shows patient data for patients undergoing vertebral body replacement including the ASA-PS (American Society of Anaesthesiologists physical status) class as well as the tumor entity

### Surgical data

All patients underwent navigated posterior instrumentation prior to surgery, a PEEK-based pedicle screw-rod system was implanted in 22 cases, while titanium implants were used in one patient treated in another hospital and in two patients due to osteoblastic metastases. Overall, 26 expandable cages with an individually adjusted size of 44 ± 14 (21–85) mm were implanted covering a median of 1 vertebra (1–4). Cage placement was performed using CT-based neuronavigation in 14 (56%) cases; in 11 cases (44%), conventional X-ray was used. Duration of surgery was 136 ± 102 (65–577) min with two cases undergoing dorsoventral instrumentation in one surgery (292 min, 577 min) and three cases of vertebrectomy. Blood loss was 800 ± 771 (100–4000) ml (Table 2). No intraoperative cage revision was necessary. Intraoperatively, one patient suffered a hemothorax leading to sudden cardiopulmonary decompensation during extubating requiring immediate surgical revision.

**Table 2:** Surgical data

Number of cages implanted • Total vertebrectomy	26 4 (15.4%)
Blood loss (ml) (Minimum–maximum)	800 ± 771 (100–4000)
Duration of surgery (min) (Minimum–maximum)	136 ± 102 (65–577)
Intraoperative imaging	
• Computed tomography • X-ray	14 (56.0%) 11 (44.0%)
Number of vertebrae instrumented by vertebral body replacement	
• 1 • 2 • 3 • 4	19 (73.1%) 5 (19.2%) 1 (3.8%) 1 (3.8%)

This table outlines data regarding levels of instrumentation as well as intraoperative data

### Clinical outcome

Regarding surgery-related complications, rebleeding was reported in 2 cases (8.0%) with two cases of hemothorax (one intraoperatively, one postoperatively) and wound healing disorders due to atrophic tissue occurred in one case (4.0%). A postoperative intensive care treatment was necessary in five cases (20.0%) (Supplementary Table 1).

Systemic infections occurred in six cases (24.0%). In detail, two cases of pneumonia and two cases of urinary tract infection were reported, as well as one patient suffering from a blood stream infection due to a catheter infection and one patient diagnosed with a bacterial superinfection of a metastasis of the shoulder (Supplementary Table 1). Furthermore, medical conditions occurred in five cases (20.0%), including pleural effusion not related to the VBR surgery in four cases (16.0%) and thromboembolic lung artery embolism as well as spontaneous thrombocytopenia after VBR in one case (4.0%) each (Supplementary Table 1). Overall, 10 patients (40.0%) developed complications postoperatively.

Regarding adjuvant tumor-specific oncological treatment, all cases were discussed in our interdisciplinary neurooncological tumor board. Thirteen patients were treated with an adjuvant combined radiochemotherapy, while five patients obtained adjuvant chemotherapy and two patients obtained adjuvant radiotherapy. No further tumor-specific treatment was performed postoperatively due to poor general condition or will of the patient in two cases, as well as due to tumor entity in one case. Two patients advised adjuvant radiochemotherapy were lost to follow-up.

**Fig. 1:**
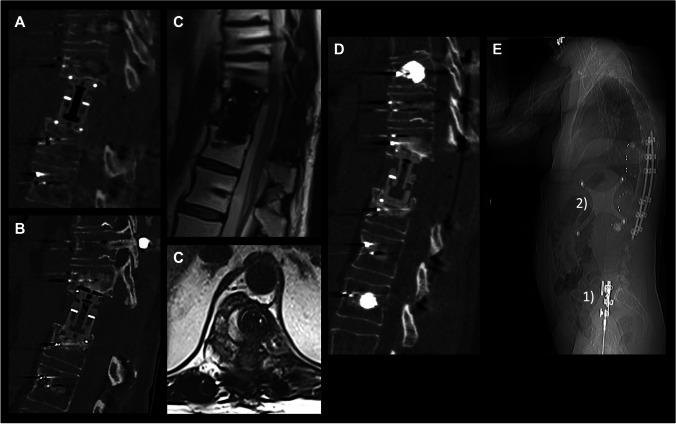
Perioperative imaging. This figure shows computed tomography imaging postoperatively (**A**) and at follow-up (**B**) with cage subsidence. Furthermore, magnetic resonance imaging (**C** = T2 in sagittal view and T2 in axial view) was acquired. Revision surgery including additional cement-augmented pedicle screw placement and cage revision (**D**) was indicated. Cage revision was performed under spinal navigation. Therefore intraoperative computed tomography imaging (**E**) was acquired, with an reference array attached to the pelvis (1) and registration matrix (2) installed to the surgical field during registration

No case of mechanic failure of the implant was observed. Surgical revision of the VBR was performed in two cases (8.0%). One patient presented 21 days postoperatively due to new ataxia. Imaging showed a fracture of the adjacent tumorous vertebrae resulting in cage loosening as well as an epidural formation. Another patient showed progressive back pain 6 months postoperatively. Imaging revealed progressive kyphosis and spinal canal stenosis due to cage subsidence (Fig. 1). Local tumor recurrence was observed in two cases 244 and 257 days after surgery.

### Radiological outcome

**Fig. 2:**
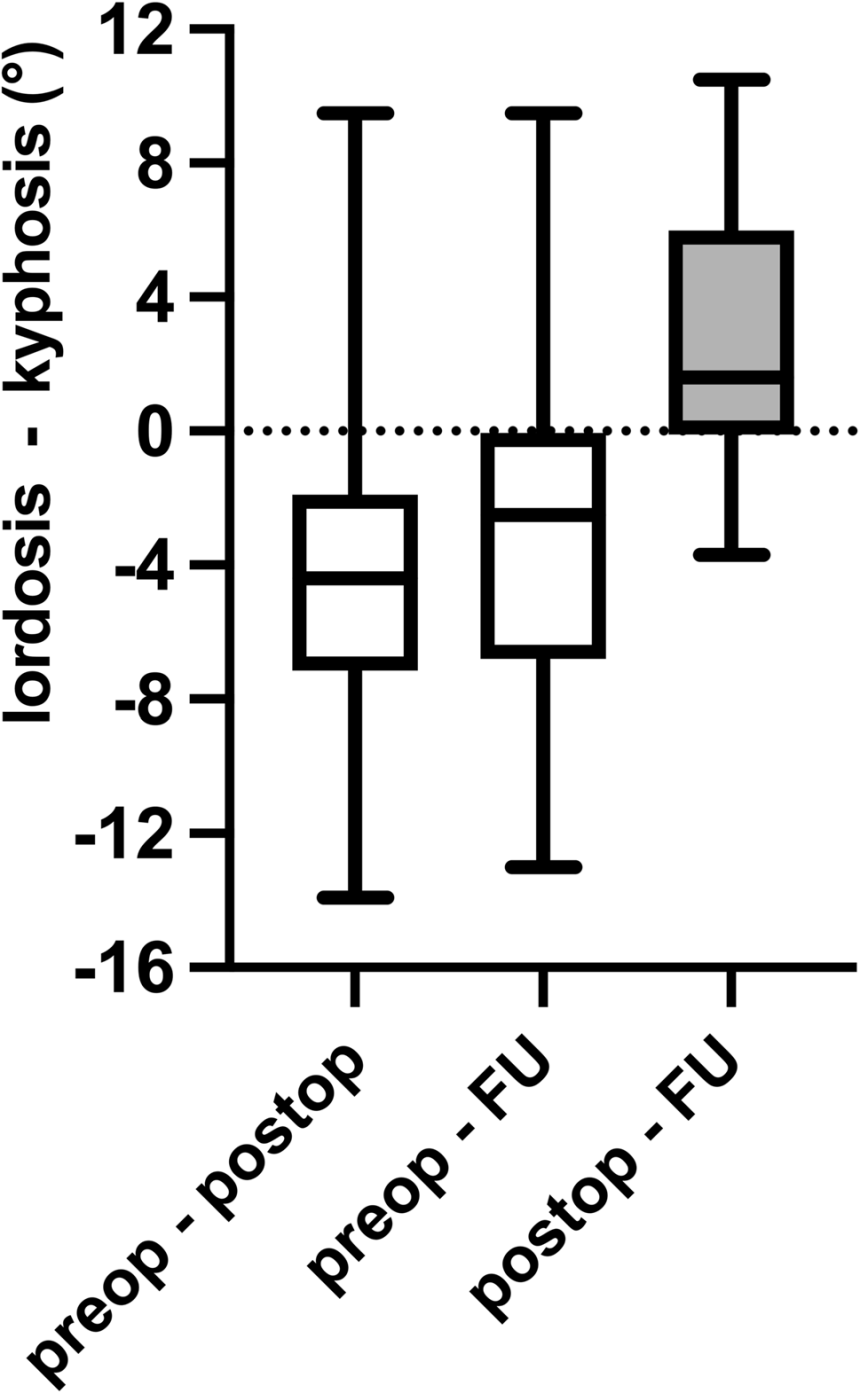
Change in the sagittal angle. This figure illustrates the change in the sagittal angle between preoperative imaging (preop), postoperative imaging (postop), and latest imaging at follow-up examination (FU). Therefore, the resulting angle between the upper endplate of the vertebrae above and upper endplate of the vertebrae below of the diseased level in a sagittal view was acquired

Radiographic analysis on preoperative and postoperative imaging was performed. Radiographic evaluation of the cages intraoperatively was sufficient in all cases, showing correct cage placement of all 26 cages. In the sagittal view, a correction of 6.3 ± 4.4 (0.2–17) degree was achieved comparing preoperative imaging and imaging after dorsoventral instrumentation (Fig. 2).

Data of radiographic follow-up was available in 17 patients for a median follow-up of 295 (13–491) days. In the sagittal view, an adaption of the sagittal angle of 5.7 ± 3.9 (0.3–13.9) degree was achieved comparing preoperative and postoperative imaging. In follow-up examination, an adaption of 4.6 ± 4.0 (0.1–13.0) degree was measured, resulting in a change in correction of 3.3 ± 3.1 (0–10.5) degree between postoperative imaging and imaging at follow-up (Fig. 2). Cage subsidence of 3.8 + 3.1 (1–8) mm was observed in six cases (35.3%) at follow-up imaging.

## Discussion

Multiple aspects must be considered choosing a suitable implant for a vertebral body replacement implant of the thoracic or lumbar spine.

High biomechanical performance is warranted as the implant has to provide mechanical stabilization and bear loads exceeding body weight by more than 250% [20]. For anterior column spine implants, mechanical stress is generally experienced by compression forces. Stability is increased by using carbon fiber to enforce the construct of the CFR PEEK VBR. Sufficient stability postoperatively is essential to enable early postoperative mobilization, which reduces postoperative complications and facilitates further oncological treatment.

Regarding the surgical procedure, no implant-related complications occurred intraoperatively regarding the CFR PEEK implant. Cage placement can be performed in a comparable manner as titanium cages, which are routinely being used. The expandable cages used in this cohort are composed of an expandable body combined with two endplates and an additional extension body if necessary. This enables an individual cage size for every patient, covering a range of 21 to 85 mm in this study.

**Fig. 3:**
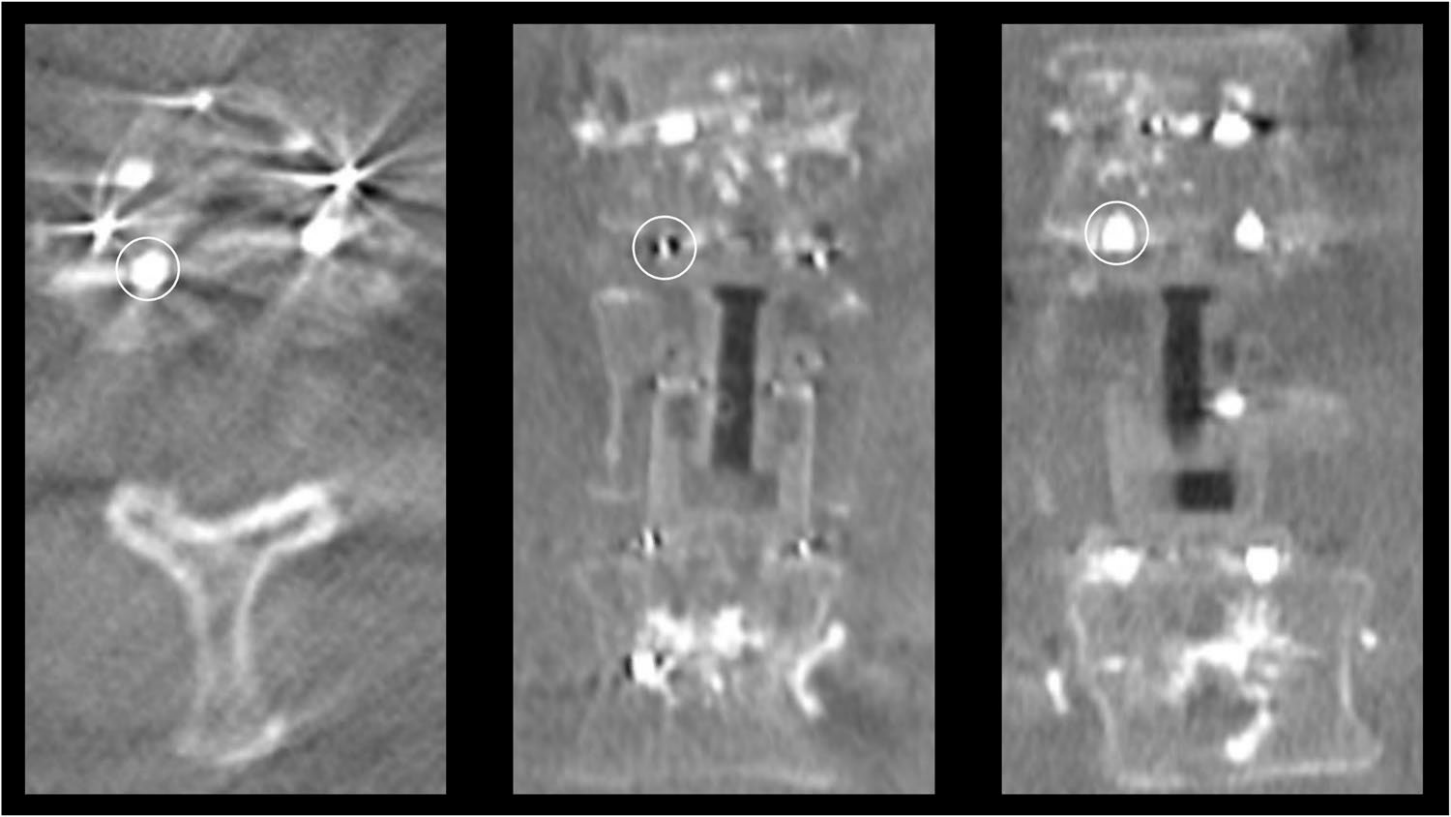
Intraoperative imaging. This figure shows intraoperative three-dimensional imaging. Titanium-based markers and pins (white circles) are integrated in the radiolucent implant for improved intraoperative visualization in X-ray imaging

Titanium-based pins and markers integrated in the radiolucent implant offer precise localization of the cage on imaging (Fig. 3). The positioning of the endplates can be clearly determined in 3D imaging as well as 2D X-ray which enables visualization of all crucial elements of the implant and clearly helps to exclude insufficient cage placement. Comparable issues as described for the assessment of CFR PEEK pedicle screw placement on intraoperative imaging were not found [7]. No case of intraoperative cage revision was required with intraoperative imaging being sufficient in all cases. Dorsoventral instrumentation resulted in a correction of 6.3 ± 4.4 (0.2–17) degree in the sagittal view. Regarding follow-up examinations, a change of 3.3 degree in the sagittal angle on average was found. No case of mechanic implant failure occurred. Cage revision was performed in one case due to a new fracture of the adjacent tumorous vertebrae and in one case due to cage subsidence.

Patients undergoing surgery due to spinal tumor manifestation typically suffer from multiple comorbidities due to their advanced age. The corresponding mean ASA-PS was three in this study. This leads to increased rates of perioperative complications as high as 40.0% in this cohort, including systemic infections, further medical conditions, and surgery-related complications. Luksanapruksa et al. reported complication rates ranging from 5.3 to 76.2% [12]. For an early and accurate detection of postoperative complications such as postoperative hematoma or infection, MRI of high quality is essential (Fig. 2).

Adjuvant stereotactic radiotherapy is necessary to provide durable local control and was performed in 60% of patients in this study [1, 18]. This requires minimal to no interference with radiotherapy (RT) planning as well as RT delivery, especially in the case of newer modalities such as proton beams, where these issues have a major impact [17]. In RT planning, target segmentation on planning CT might be impaired due to implants. Image artifacts might reduce the quality of the scan, potentially obscure target and risk structures, and lead to inaccuracies regarding image fusion [6, 11]. Furthermore, CT artifacts surrounding metallic implants lead to inaccurate estimation of Hounsfield Units and electron density, resulting in dose calculation errors [10, 11]. Regarding RT delivery, the high electron density of metallic implants leads to dose perturbation [11, 13]. This might result in compromised tumor control of structures located distal to the implant and lead to overdosing of potential adjacent risk structures such as the spinal cord [11, 13]. CFR PEEK–based implants show reduced artifacts on CT imaging and help to minimize effects of dose perturbation in RT delivery [11, 13, 14]. This has been mainly investigated for CFR PEEK–based pedicle screws; however, technical considerations have been made regarding CFR PEEK–based implants in general [11, 14, 22]. Effects like CT artifacts and dose perturbation surrounding metallic implants are mostly related to the size and location of the implant; the benefits found in CFR PEEK–based pedicle screws and other implants can therefore also be transferred to CFR PEEK VBR implants [11, 14, 22].

Furthermore, MRI is the main imaging modality, used in the detection of tumor recurrence. Minimizing interference of the implant with MRI using CFR PEEK subsequently improves early detection of tumor recurrence (Fig. 1). Metal artifact reduction sequences (MARS) have been developed aiming to reduce metal-induced artifacts [9, 15]. However, MARS leads to increased scanning time and reduced image resolution and the availability is limited [8].

## Conclusion

CFR PEEK implants of the thoracic and lumbar spine enable advantageous postoperative surveillance imaging and effective delivery of postoperative adjuvant radiotherapy. Our data confirms that CFR PEEK VBR represents a legitimate surgical strategy if dorsoventral instrumentation for construct stability is needed in patients suffering from spinal tumor manifestations.

## Supplementary Information

Below is the link to the electronic supplementary material.

Supplementary file1 (DOCX 17 kb)

## Data Availability

The datasets generated during and/or analyzed during the current study are available from the corresponding author on reasonable request.

## Abbreviations

3D: Three-dimensional
ASA-PS: American Society of Anaesthesiologists physical status
CFR PEEK: Carbon fiber–reinforced polyetheretherketone
CT: Computed tomography
MARS: Metal artifact reduction sequences
MRI: Magnetic resonance imaging
RT: Radiotherapy
VBR: Vertebral body replacement
