# Supplementary material for: Clinical evaluation of vertebral body replacement of carbon fiber–reinforced polyetheretherketone in patients with tumor manifestation of the thoracic and lumbar spine
Source: Acta Neurochir (Wien). 2023 Feb 23;165(4):897–904. doi: 10.1007/s00701-023-05502-z (PMC10068665; doi:10.1007/s00701-023-05502-z)
Supplement: Supplementary file 1 — Supplementary file1 (DOCX 17 kb) [file 701_2023_5502_MOESM1_ESM.docx]

| **n (%)** |  |
| --- | --- |
| **Complications overall** | **10 (40.0)** |
| **Surgery-related complications**   - **Hemothorax**   - intraoperatively   - postoperatively - **Atrophic wound healing disorder** | **3 (12.0)**  **2 (8.0)**  1 (4.0)  1 (4.0)  **1 (4.0)** |
| **Intensive care unit treatment** | **5 (20.0)** |
| **Systemic infections**   - Urinary tract infection - Pneumonia - Blood stream infection - Superinfection of a metastasis of the shoulder | **6 (24.0)**  2 (8.0)  2 (8.0)  1 (4.0)  1 (4.0) |
| **Further medical conditions**   - Pleural effusion - Thrombocytopenia - Lung artery embolism (thromboembolic) | **5 (20.0)**  4 (16.0)  1(4.0)  1(4.0) |

**Supplementary Table 1: Postoperative complications**

This table lines up the rate of postoperative complications. All infections requiring systemic antibiotic treatment, excluding wound infections were considered as systemic infections.
